# Effects of low-intensity resistance exercise with blood flow restriction after high tibial osteotomy in middle-aged women

**DOI:** 10.1097/MD.0000000000032294

**Published:** 2022-12-23

**Authors:** Han-Soo Park, Jun-Seob Song, Eun-Kuk Kim

**Affiliations:** a Korean National Sports University, Songpa-gu, Seoul, Republic of Korea; b Gangnam JS Hospital, Gangnam-gu, Seoul, Republic of Korea; c SRC Hospital, Chowol-eup, Gwangju-si, Gyeonggi-do, Republic of Korea.

**Keywords:** arterial occlusion pressure, blood flow restriction, high tibial osteotomy, osteoarthritis, postoperative rehabilitation

## Abstract

High tibial osteotomy (HTO) is an effective surgical method for treating medial compartment osteoarthritis. However, in most cases after surgery, muscle strength is decreased, and rapid muscle atrophy is observed. Therefore, the purpose of this study is to verify the effects of low-intensity resistance exercise (LIE) with blood flow restriction (BFR) on the cross-sectional area (CSA) of thigh muscles, knee extensor strength, pain, and knee joint function and investigate proper arterial occlusion pressure (AOP) in middle-aged women who underwent HTO.

**Method:**

This study was designed as a prospective randomized controlled trial. Forty-two middle-aged women who underwent HTO were randomly divided into three groups and participated in LIE with (40% or 80% AOP applied) or without BFR. The main outcome was the measurement of the CSA of thigh muscles (at 30% and 50% distal length of the femur) before and 12 weeks after treatment. Additionally, knee extension muscle strength, pain, and joint function were evaluated before and 6 and 12 weeks after treatment.

**Results:**

CSA of thigh muscles at 30% and 50% distal length of the femur decreased in the AOP 40% and control groups and was the largest in the AOP 80% group 12 weeks after treatment. Knee extension strength increased in all groups and was the highest in the AOP 80% group 6 and 12 weeks after treatment. Pain improved in all groups, with no intergroup differences. Knee joint function improved in all groups and was superior in the 80% AOP group 12 weeks after treatment.

**Conclusion:**

LIE with BFR at 80% AOP was effective in preventing atrophy of the thigh muscle, increasing muscle strength, and improving function. BFR at 40% AOP had no difference in the results when compared with the group in which BFR was not applied. Therefore, LIE with an AOP of 80% is recommended for patients undergoing HTO.

## 1. Introduction

Approximately 240 million people worldwide are diagnosed with knee osteoarthritis (OA) annually.^[1]^ Knee arthritis is more common and severe in women than in men, and is accompanied by more severe functional decline.^[2–5]^ High tibial osteotomy (HTO), a surgical method for treating medial compartment osteoarthritis (MCOA), improves symptoms by moving the mechanical axis from the medial to the lateral side of the knee joint and is more effective in middle-aged patients than in older patients.^[6–11]^ Although HTO can improve MCOA symptoms, thigh muscle strength is also crucial for restoring the joint function and maintaining patient satisfaction with the surgery.^[12]^ However, in most patients, muscle strength decreases after surgeries, and rapid muscle atrophy occurs owing to preoperative joint symptoms, pneumatic tourniquet use during surgery, and implementation of a non-weight-bearing phase after surgery.^[13–16]^ This decreased muscle strength and muscle atrophy can last for several years and lead to a decrease in the knee joint function, thereby restricting daily life activities and participation in sports and increasing the risk of falls, which can cause serious complications.^[17–21]^

High-intensity resistance exercise is effective in improving muscle strength and atrophy (60–100% of 1-repetition maximum [RM]).^[22]^ However, it is not suitable for postoperative patients, as patients require time for recovery and it increases pain at the surgical site and load on the joint.^[21]^ Therefore, in some recent studies, low-intensity resistance exercise (LIE) with blood flow restriction (BFR) has been suggested as an alternative to high-intensity resistance exercise.^[23–26]^ LIE with BFR blocks venous return during the performance of resistance exercise at 20% to 30% of 1 RM while maintaining arterial inflow to the muscle by applying pressure to the limb through the use of tourniquets.^[10]^

Although muscle hypertrophy resulting from this method has not yet been completely explained, some studies suggest that this method increases muscle protein synthesis by increasing cellular signals and hormones, leads to the proliferation of muscle progenitor cells, increases anabolic and anticatabolic signals, and stimulates recruitment of type II muscle fibers.^[3,27–33]^ However, studies on the application of this therapy in patients who underwent surgery for OA are few.^[23,34–37]^ In addition, for healthy people, applying 80% arterial occlusion pressure (AOP) has been more effective than applying 40% AOP; however, effective AOP for patients undergoing OA surgery has not been suggested.^[38]^ Therefore, this study aimed to investigate the effects of LIE with BFR and determine the appropriate AOP for patients who underwent HTO for treatment of MCOA.

## 2. Methods

### 2.1. Participants and study design

This study included middle-aged women (45–65 years old) who underwent HTO performed by a single surgeon at an orthopedic hospital (Gangnam JS Hospital, Gangnam, Seoul, Korea). Patients with body mass index (BMI) > 30 kg/m^2^, severe hypertension (>170/110 mm Hg), deep venous thrombosis, endothelial dysfunction, peripheral vascular disease, diabetes, or orthopedic injuries to the lower limbs were excluded.

### 2.2. Randomization, masking, and blinding

The recruited participants were randomly allocated to the 80% AOP (BFR at 80% AOP was applied), 40% AOP (BFR at 40% AOP was applied), and control (no BFR was applied) groups. For each group assignment randomization, an unmarked envelope containing a random number was handed out to patients by a therapist who was not involved in this study during the consultation. To reduce bias, all the participants in this study were blinded to group assignment (experimental or control group.

One of the investigators (H.S.) was not blinded because he helped apply the interventions to the participants, but he was blinded to the evaluation of the results and statistics of the study. All evaluations of the study were conducted by radiologists and therapists who did not participate in the study, and the 2 investigators (E.K. and J.S.) who performed the evaluations were blinded to the assignment of participants and the interventions provided by the therapist.

### 2.3. LIE protocol

The LIE protocol was divided into a 6-week non-weight-bearing phase and a 6-week full-weight-bearing phase.^[39]^ All participants were administered the same LIE protocol at the Sports Medical Center of our hospital, and participated in the exercise program twice a week for 12 weeks. Because it is difficult for participants to measure 1RM, the OMNI resistance exercise scale was used to set a patient’s exercise intensity to 30% of 1 RM (0, extremely easy; 10, extremely hard).^[40]^ The exercise intensity of all groups was measured in the range of 1 to 3 on the OMNI-resistance exercise scale.^[41]^ BFR was applied to quadriceps setting exercise, straight leg raise, and knee extension, and hamstring curl using a Thera-band, and applied to leg extension and hamstring curl using a machine, leg press, squat, and lunge after 6 weeks. For all exercises to which BFR was applied, 4 sets of 30/15/15/15 repetitions were performed. Table 1 shows the exercise protocol and application of LIE with BFR.

**Table 1:** Exercise protocol.

Phase	Classification	Exercise	Application of BFR	Repetition or time
NWB phase (0–6 wks)	Warm-up	UBC	X	15 min
		Stretching	X	
		ROM exercise	X	
	Strengthening	Q/H setting	O	30/15/15/15
		Four-way SLR	O	30/15/15/15
		Knee extension with Thera-band	O	30/15/15/15
		Hamstring curl with Thera-band	O	30/15/15/15
	Additional exercise	Ankle dorsi/plantar flexion with Thera-band	X	10 min
		Hip ab/adduction	X	5 min
	Cool down	Cool down	X	
FWB phase (6–12 wks)	Warm-up	Stationary Bike	X	15 min
		Stretching	X	
	Strengthening	Leg extension with machine	O	30/15/15/15
		Hamstring curl with machine	O	30/15/15/15
		Leg press	O	30/15/15/15
		Squat	O	30/15/15/15
		Lunge	O	30/15/15/15
	Additional exercise	Balance & proprioceptive exercise	X	10 min
	Cool down	Cool down	X	5 min

BFR = Blood flow restriction, FWB = Full-weight bearing, NWB = Non-weight bearing; UBC, Upper body cycle, Q/H = Quadriceps/hamstring, ROM = Range of motion, SLR = Straight leg raises.

### 2.4. AOP procedure

**Figure 1.**
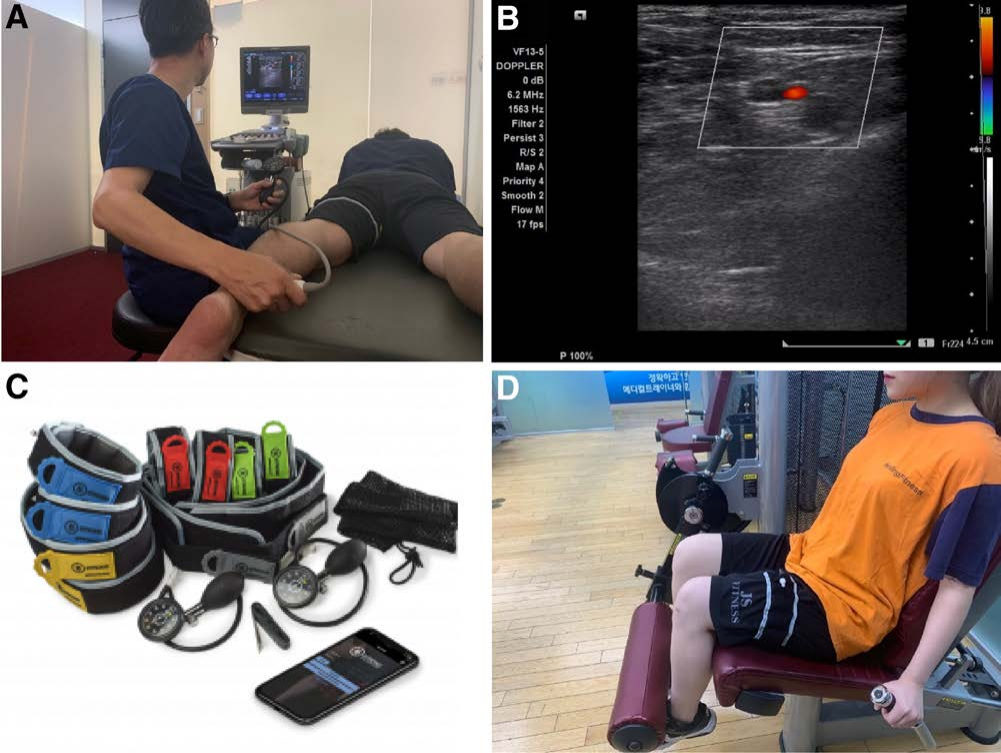
Application of the blood flow restriction. (A) Measurement of arterial occlusive pressure using Doppler ultrasound. (B) Pulsation of the posterior tibial artery. (C) Strong training system^TM^ for blood flow restriction. (D) Applying a tourniquet to restrict blood flow to the proximal femur.

AOP was measured when the posterior tibial artery pulse disappeared upon slowly applying pressure with a pneumatic tourniquet (B Strong, Parksity, Utah) worn on the proximal thigh. Doppler ultrasound (ACUSON SC2000, Siemens Medical Solutions Inc., MV) was used to palpate the pulse (Fig. 1A). After AOP measurement, 40% or 80% AOP was applied using a pneumatic tourniquet to partially occlude the artery with vein occlusion, and the pulse was palpated again (Fig. 1B). If the pulse was not confirmed, AOP was remeasured after 10 minutes of rest. The pneumatic tourniquet was worn on the proximal thigh and maintained constant pressure during resistance exercise (Fig. 1C, D). The personalization of AOP not only restricted blood flow to the veins and partially maintained arterial blood flow but also allowed patients to safely apply BFR, regardless of thigh circumference, cuff size, and systolic blood pressure.^[42]^ In this study, sets of BFR for 5 minutes were followed by reperfusion for 3 minutes. During exercise, pulse was checked through palpation of the posterior tibial artery to measure a patient’s blood flow. If the exercise was accompanied by physical discomfort, such as thigh pain, numbness, or tingling in the lower extremities, it was stopped.^[43]^

### 2.5. Measurement of cross-sectional area of the thigh

**Figure 2.**
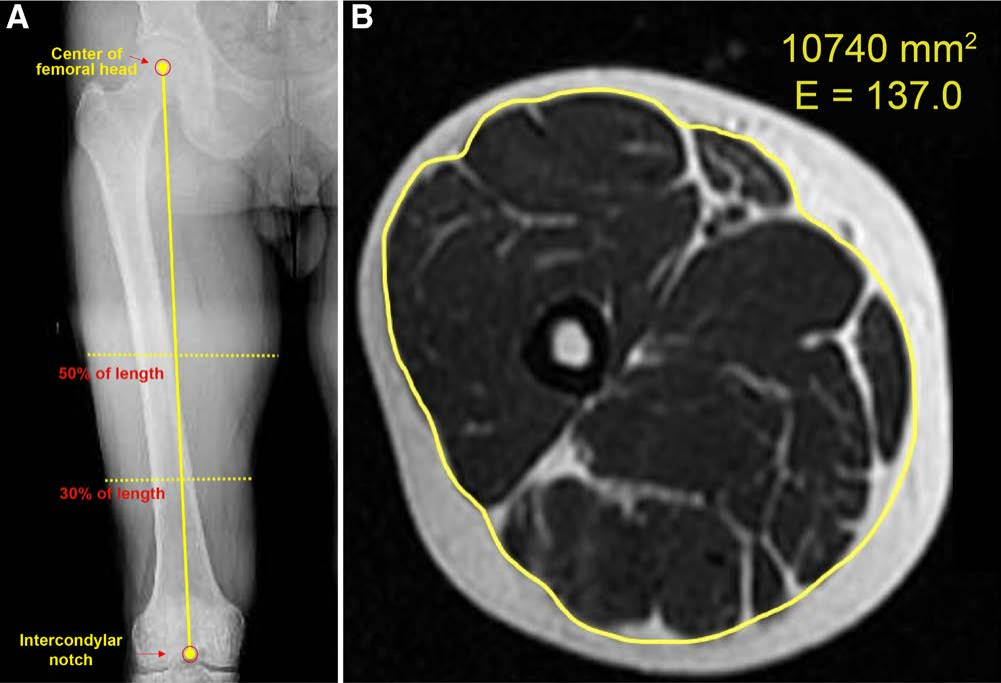
(A) Confirmation of the measurement of the thigh cross-sectional area location using full-length X-ray. (B) Measurement of the cross-sectional area of the thigh at 50% of the distal femur with the free ROI technique in the PACS program. ROI = region of interest.

The primary outcome of this study was changes in the thigh muscles after treatment. To measure these changes, the cross-sectional area (CSA) of the thigh was measured using magnetic resonance imaging (MRI; Chorus 1.5-T MRI, ISOL, Gwangju, Korea) before and at 12 weeks after treatment. *T*2-weighted axial images of 8-mm slices were obtained, with a repetition time of 6120, an echo time of 119, a field of view of 400, and a matrix size of 512 × 512. CSA of the thigh was assessed by measuring the center of the femoral head and the length of the intercondylar notch using a full-length radiograph of the thigh and confirming the distal 30% and 50% points (Fig. 2A). A radiologist not involved in the study used an 800-dpi mouse to measure the CSA of the thigh muscle by drawing along the fascia of the muscle using the free region of interest (ROI) technique of the PACS program (Viewrex, HechEhim, Guro, Korea) (Fig. 2B). Each value was measured twice and was used when the Cronbach’s alpha value was α ≥ .8 through reliability analysis.^[38]^

### 2.6. Measurement of knee extensor strength

The second primary outcome measure was knee extensor strength. The maximum voluntary isometric contraction of the knee joint extensor along with the CSA of the thigh was measured using an isokinetic dynamometer (HUMAC Norm; CSMi, Stoughton, MA) before and at 6 and 12 weeks after treatment. This test was performed at 60° of knee flexion to measure the strength of the knee extensors. maximum voluntary isometric contractions were measured after 10 seconds of rest after 2 sub-maximum contractions at 60° of knee flexion that were each performed for 5 seconds, with 5- seconds breaks in between. The measurement value is expressed as a percentage (%) of the peak torque/body weight value.^[44,45]^

### 2.7. Assessment on pain and function of the knee joint

Changes in knee pain and function, the secondary outcomes of this study, were evaluated before and 6 and 12 weeks after treatment using questionnaires. Pain was assessed using a visual analog scale, and function was assessed using the International Knee Documentation Committee questionnaire.

### 2.8. Statistical analysis

Data were analyzed using the SPSS program (version 23.0; IBM Corp., Somers, NY). All data are expressed as mean ± standard deviation. Before verifying intergroup and intragroup comparisons, normality verification was performed using the Shapiro–Wilk test. The Wilcoxon signed rank test was performed to compare before and after values of each independent variable. To verify differences among groups, age and BMI were analyzed using 1-way analysis of variance. The Kruskal–Wallis test was performed for variables not satisfying normality. For post hoc analysis of the Kruskal–Wallis test, the Bonferroni adjustment method was used after the Mann–Whitney *U* test. A simple regression analysis was performed to analyze the relationship of thigh CSA and knee muscle strength on pain and knee joint function. All statistical significance levels were set at *α* < .05.

### 2.9. Ethical considerations

All participants were given complete information on the risks, discomforts, and purpose of the study protocol before their participation, and they provided institutionally approved written informed consent. The study protocol was approved by the Institutional Review Board of the Korea Ministry of Health and Welfare (P01-202005-11-002).

## 3. Results

**Figure 3.**
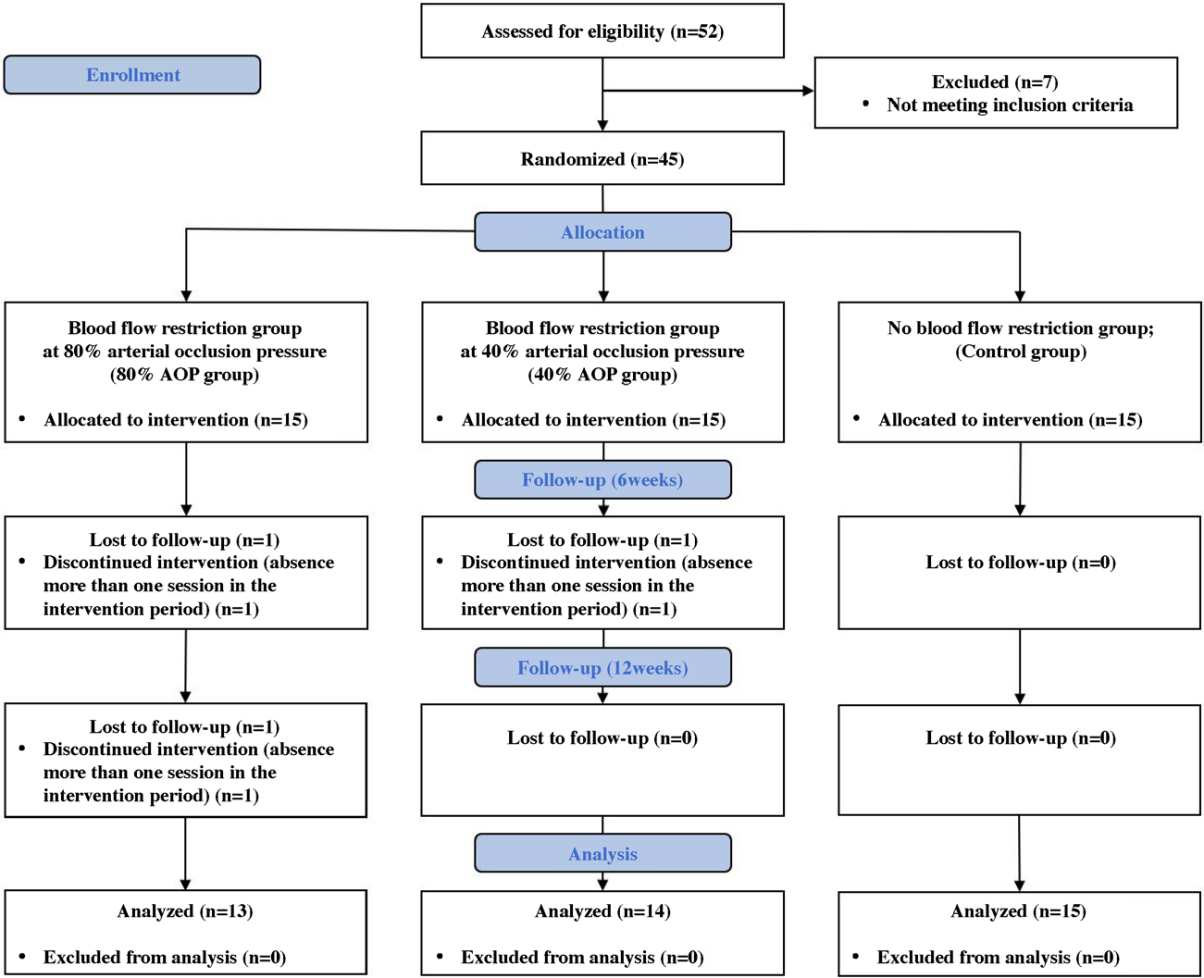
The CONSORT flow diagram.

A flow diagram of the study process is shown in Figure 3. Forty-five participants were enrolled in this study and were divided into 3 groups of 15 people per group. During the study, 2 participants in the 80% AOP group and 1 participant in the 40% AOP group were lost to follow-up; thus, the final analysis included 13 (58.7 ± 1.2) in the 80% AOP group, 14 (59.8 ± 1.2) in the 40% AOP group, and 15 (57.5 ± 1.3) in the control group (Fig. 3). The participant characteristics according to the groups are presented in Table 2.

**Table 2:** Characteristics of the participants^a^ (n = 42).

	80% AOP group (n = 13)	40% AOP group (n = 14)	Control (n = 15)	*P* value
Age, yrs	58.7 ± 1.2	59.8 ± 1.2	57.5 ± 1.3	.505^b^
Height, cm	160 ± 5.1	156.9 ± 4.6	157.6 ± 2.8	.150^c^
Weight, kg	62.3 ± 3.4	60.7 ± 7.2	62.7 ± 4.2	.576^c^
BMI, kg/m^2^	24.5 ± 0.5	24.6 ± 0.6	25.2 ± 0.5	.620^b^
Side				
Right, n	7	9	6	
Left, n	6	5	9	

a All data are expressed as mean ± standard deviation.

b Kruskal–Wallis test.

c One-way ANOVA.

AOP = arterial occlusion pressure, BMI = body mass index.

### 3.1. CSA of the thigh

**Figure 4.**
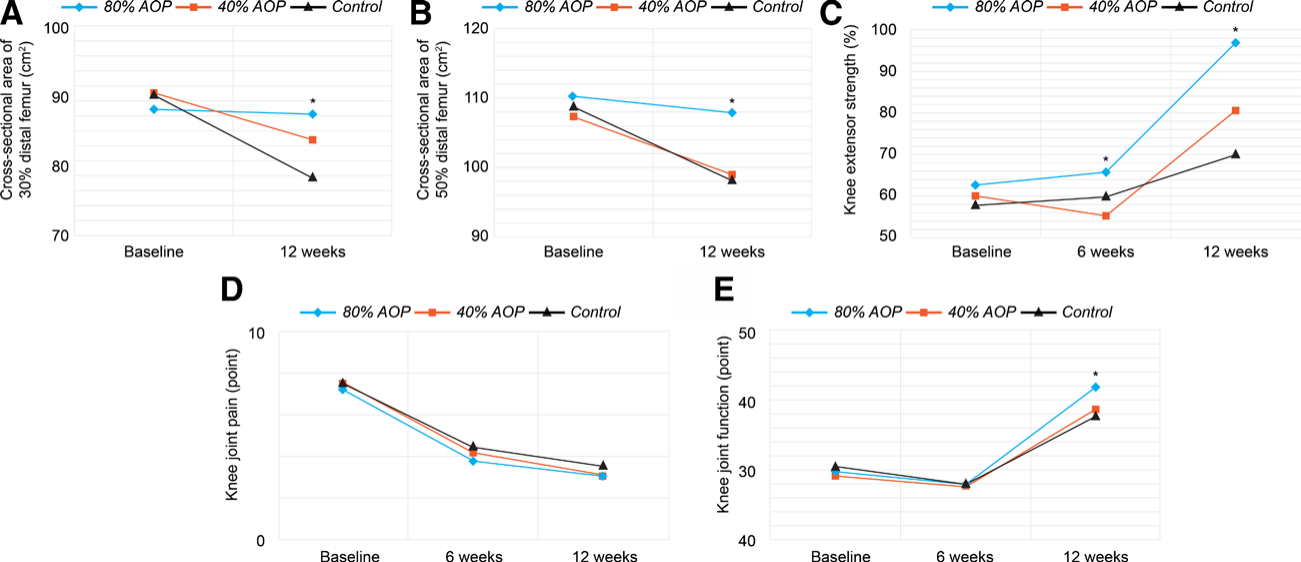
These graphs show the following results. (A) Cross-sectional area of 30% distal femur (Post hoc: 80% AOP > Control, 80% AOP = 40% AOP, 40% AOP = Control). (B) Cross-sectional area of 50% distal femur (Post hoc: 80% AOP > 40% AOP = Control). (C) Knee extensor strength (Post hoc at 6 weeks and 12 weeks: 80% AOP > 40% AOP = Control). (D) Pain score. (E) Function score (Post hoc: 80% AOP > 40% AOP = Control). (*: *P < *.05 by the Kruskal–Wallis test). AOP = occlusion pressure.

CSA of the thigh at 30% of the distal femur changed from 88.2 ± 6.6 cm^2^ pretreatment to 87.5 ± 5.7 cm^2^ post-treatment in the 80% AOP group (*P = *.442), decreased from 90.5 ± 7.7 cm^2^ pretreatment to 83.8 ± 8.1 cm^2^ post-treatment in the 40% AOP group (*P = *.003), and decreased from 90.3 ± 7.4 cm^2^ pretreatment to 78.2 ± 7.22 post-treatment in the control group (*P = *.001). CSA of the thigh at 50% of the distal femur changed from 110.1 ± 8.5 cm^2^ pretreatment to 107.9 ± 5.7 cm^2^ post-treatment in the 80% AOP group (*P = *.184), decreased from 107.5 ± 7.7 cm^2^ pretreatment to 98.9 ± 7.7 cm^2^ post-treatment in the 40% AOP group (*P = *.003), and decreased from 108.8 ± 8 cm^2^ pretreatment to 98.2 ± 8.7 cm^2^ post-treatment in the control group (*P = *.001). There was no significant difference in thigh CSA at 30% and 50% of the distal femur among the groups pretreatment; however, post-treatment, the 80% AOP group showed a larger CSA than the other 2 groups (*P = *.005, *P* = .012, respectively) (Fig. 4A, B).

### 3.2. Knee extension muscle strength

Increase in knee extension muscle strength in the 80% AOP group from 62.6%±10.4% pretreatment to 65.5%±10.4% at 6 weeks post-treatment was not significant (*P = *.344); however, it increased significantly to 96.8%±12% at 12 weeks post-treatment (*P < *.001). In the 40% AOP group, it did not change significantly, from 60.1%±10.8% pretreatment to 55.3%±11.1% at 6 weeks post-treatment (*P = *.108), but increased significantly to 80.6%±20.1% at 12 weeks post-treatment (*P < *.001). In the control group, it did not change significantly, from 57.9%±8.7% pretreatment to 59.9%±10% at 6 weeks post-treatment (*P = *.733), but increased significantly to 78.1%±12.4% at 12 weeks post-treatment (*P < *.001). There was no statistically significant difference among the groups pretreatment (*P = *.395), but knee extension strength in the 80% AOP group was higher than that in the other 2 groups at 6 and 12 weeks post-treatment (*P < *.05 for all) (Fig. 4C).

### 3.3. Knee pain and joint function

Pain scores in the 80% AOP group decreased from 7.3 ± 1.1 pretreatment to 3.8 ± 1.6 at 6 weeks post-treatment (*P < *.001), with no significant change to 3.1 ± 1.3 at 12 weeks post-treatment (*P = *.111). Pain in the 40% AOP group decreased from 7.5 ± 1.4 pretreatment to 4.2 ± 1.6 and 3.1 ± 1.2 at 6 and 12 weeks post-treatment, respectively (*P < *.05 for all). Pain in the control group decreased from 7.5 ± .9 pretreatment to 4.4 ± 1.5 and 3.5 ± 1.2 at 6 and 12 weeks post-treatment, respectively (*P < *.05 for all). There was no significant difference in pain levels among the groups (*P > *.05) (Fig. 4D). Knee joint function in the 80% AOP group did not change significantly, from 29.7 ± 5.5 pretreatment to 28.1 ± 3.2 at 6 weeks post-treatment (*P = *.116), but increased significantly to 42.1 ± 3.5 at 12 weeks post-treatment (*P < *.001). Function in the 40% AOP group did not change significantly, from 29.1 ± 7.4 pretreatment to 27.6 ± 4.1 at 6 weeks post-treatment (*P = *.263), but increased to 38.9 ± 3.3 at 12 weeks post-treatment (*P < *.001). Function in the control group decreased from 30.4 ± 5.4 pretreatment to 27.9 ± 4.5 at 6 weeks post-treatment (*P = *.007) and increased to 38.3 ± 3.8 at 12 weeks post-treatment (*P* < .001). There was no significant difference in knee joint function 6 weeks post-treatment among the groups (*P* = .917), but the 80% AOP group had the highest score (*P = *.033) at 12 weeks post-treatment (Fig. 4E).

### 3.4. Effects of CSA of the thigh muscle and knee extension strength on knee pain and function

CSA of the thigh muscle at 30% and 50% of the distal femur did not affect knee pain pre- or post-treatment (*P > *.05) or knee joint function pretreatment. However, CSA of the thigh muscle was related to joint function post-treatment, which increased with increasing CSA (*R*^2^ = .156, *R*^2^ = .108; *P < *.05 for both). Knee extension strength influenced pretreatment pain, with lower strength correlating with higher pain (*R*^2 ^= 0.261; *P = *.001). However, strength was not associated with post-treatment pain (*P = *.141). Knee extension strength was not associated with pretreatment joint function (*P = *.109) but it was related to post-treatment function, with function increasing as the strength increased (*R*^2^ = 579; *P < *.001) (Table S1, Supplemental Digital Content, http://links.lww.com/MD/I126).

## 4. Discussion

According to the results of this study, in middle-aged women who underwent HTO, 80% AOP was effective in preventing thigh muscle atrophy, increasing knee extensor strength, and improving function compared with other approaches. Interestingly, in all the results, there was no significant difference between the 40% AOP group and the control group, and the CSA of the thigh muscle was decreased, despite participating in LIE.

In patients with OA, pain is alleviated after surgery, but circumference and strength of thigh muscles are reduced, leading to decreased knee joint function and increased risk of falls.^[46,47]^ In a study similar to ours, Dreyer et al^[13]^ measured the reduction in CSA of thigh muscles after total knee replacement using MRI and found that CSA decreased by 1% per day for 2 weeks, with a total CSA reduction of 18% by 6 weeks.^[13]^ This can reduce anabolic reactions that synthesize muscle proteins, allowing for catabolic reactions to degrade the proteins more actively, thereby reducing muscle mass in the thigh.^[18]^ In our study, CSA of the thigh muscle decreased at 12 weeks post-surgery in all groups, except in the 80% AOP group. This corroborates the findings of Takarada et al,^[48]^ who reported that the group in which BFR was applied showed a smaller decrease in thigh CSA compared to the group that did not use BFR. In addition, application of 80% AOP was the most effective.^[48]^ In a study by Lixandrão et al,^[38]^ BFR was applied to participants with 40% and 80% AOP, and thigh CSA was measured using MRI. They reported that the group that applied 80% AOP with an exercise intensity of 40% of 1 RM had the greatest increase in both thigh muscle strength and CSA.^[38]^ In addition, Dephillipo et al^[42]^ reported that, in both healthy people and patients who underwent arthroscopy, when performing BFR, applying 80% AOP effectively resulted in muscle hypertrophy.^[42]^ Therefore, our results and other findings in the literature show that applying 80% AOP was the most effective in achieving muscle hypertrophy and preventing muscle atrophy.

Applying 80% AOP was also effective in increasing knee extension strength. According to our results, all groups showed an increase at 12 weeks post-treatment, with the AOP 80% group showing the best results. There was no increase in strength until more than 6 weeks after treatment in any of the groups, which is most likely because resistance exercise generally results in muscle hypertrophy after an increase in strength, but strength follows hypertrophy when LIE is applied with BFR.^[36]^ Some researchers believe that general resistance exercise results in an adaptation to exercise, where neurological adaptations such as the increased recruitment of motor units occur first, leading to an increase in muscle strength before hypertrophy. Neurological adaptation is believed to occur late in BFR, resulting in hypertrophy before strength.^[49,50]^ In addition to our results showing increased muscle strength with BFR, there are extensive data from similar studies that corroborate our findings. Hughes et al^[51]^ compared a high-intensity resistance exercise group (exercise intensity of 70% of 1 RM) with a LIE with a BFR group (exercise intensity is 30% of 1 RM) in 28 patients who underwent anterior cruciate ligament reconstruction, and found that LIE with BFR was more effective in increasing muscle hypertrophy and muscle strength than high-intensity resistance exercise. High-intensity resistance exercise in general is effective in increasing muscle hypertrophy and muscle strength^[22]^; however, in patients who find high-intensity resistance exercise challenging post-surgery, application of LIE with BFR is beneficial as it can minimize load on the joint and protect the surgical site. Therefore, we believe that the application of BFR after HTO can safely and effectively increase muscle strength and hypertrophy.

Pain improved in all groups, with no significant difference among the groups. In addition, CSA of the thigh pre- and post-treatment, and muscle strength post-treatment did not affect pain. We believe that this pain relief effect is because HTO moved the mechanical axis of the medial compartment of the knee to the lateral compartment.^[52,53]^ Our results also show that a patient’s initial muscle strength influences pretreatment pain. Bokaeian et al^[54]^ reported that pain is remarkably correlated with muscle strength in patients with OA and that weakened muscle strength can aggravate OA symptoms. This suggests that increasing muscle strength is necessary to reduce pain in patients with OA without surgery.^[54]^

Knee joint function improved as CSA and muscle strength of the thigh increased, with the highest function observed in the AOP 80% group. Our results showed that although pain caused by OA can be improved through HTO, hypertrophy of thigh muscles and increase in muscle strength are essential for improving function.

Despite the significant findings, our study had some limitations. First, the number of participants in our study was small, and the follow-up period was short. Second, we determined muscle strength by measuring the isometric strength of the knee joint at a single angle, excluding possible values of knee flexion and strength at other angles. Finally, we assessed the patients’ knee joint function using a questionnaire, making it difficult to accurately evaluate functions. Therefore, a more structured functional evaluation, including balance ability, and a more comprehensive measurement of thigh muscle strength are needed in the future.

## 5. Conclusions

In middle-aged women who received HTO for the treatment of MCOA, the application of BFR at 80% AOP is effective in preventing atrophy of the thigh muscle, increasing muscle strength, and improving function. Therefore, we recommend BFR at 80% AOP during rehabilitation of patients who undergo OA. In addition, LIE with BFR may contribute to postoperative pain reduction and improvement in function safely and effectively, as it does not burden the operated joint, unlike high-intensity exercises. These results demonstrate the benefit of BFR therapy as a postoperative treatment and suggest a need to adapt this therapy for more surgeries because it can effectively protect muscle strength while protecting the joint post-surgery.

## Acknowledgments

We would like to thank Editage (www.editage.co.kr) for English language editing.

## Author contributions

**Conceptualization:** Han-Soo Park, Jun-Seob Song.

**Data curation:** Han-Soo Park.

**Formal analysis:** Han-Soo Park, Eun-Kuk Kim.

**Writing – original draft:** Han-Soo Park, Eun-Kuk Kim.

**Writing – review & editing:** Han-Soo Park, Eun-Kuk Kim.

## Supplementary Material
