# Supplementary material for: Effects of low-intensity resistance exercise with blood flow restriction after high tibial osteotomy in middle-aged women
Source: Medicine (Baltimore). 2022 Dec 23;101(51):e32294. doi: 10.1097/MD.0000000000032294 (PMC9794348; doi:10.1097/MD.0000000000032294)
Supplement: Supplementary file 1 [file medi-101-e32294-s001.pdf]

Supplemental Digital Content Table 1: Effects of the cross-sectional area of the thigh muscle and knee extension strength on knee pain and function[a]

	CSA of thigh at distal 30%		CSA of thigh at distal 50%		Knee extension strength	
	R^2	*P* value	R^2	*P* value	R^2	*P* value
Pretreatment pain	<.001	.972	.025	.318	.261	<*.001******
Posttreatment pain	.005	.649	.024	.330	.053	.141
Pretreatment function	.020	.373	.006	.620	.063	.109
Posttreatment function	.156	*.010**	.108	.034	.579	<*.001******

CSA: cross-sectional area

[a]Simple regression analysis = *: <0.05, ***: 0.01
